# Bioturbation Intensity Modifies the Sediment Microbiome and Biochemistry and Supports Plant Growth in an Arid Mangrove System

**DOI:** 10.1128/spectrum.01117-22

**Published:** 2022-06-01

**Authors:** Marco Fusi, Jenny Marie Booth, Ramona Marasco, Giuseppe Merlino, Neus Garcias-Bonet, Alan Barozzi, Elisa Garuglieri, Tumeka Mbobo, Karen Diele, Carlos M. Duarte, Daniele Daffonchio

**Affiliations:** a Division of Biological and Environmental Science and Engineering (BESE), Red Sea Research Center (RSRC), King Abdullah University of Science and Technology (KAUST), Thuwal, Saudi Arabia; b Centre for Conservation and Restoration Science, School of Applied Sciences, Edinburgh Napier University, Edinburgh, United Kingdom; c Coastal Research Group, Department of Zoology and Entomology, Rhodes University, Grahamstown, South Africa; d Computational Bioscience Research Center (CBRC), King Abdullah University of Science and Technology (KAUST), Thuwal, Saudi Arabia; e National Research Foundation-South African Institute for Aquatic Biodiversity Institute, Makhanda, South Africa; f South African National Biodiversity Institute, Kirstenbosch Research Centre, Cape Town, South Africa; g Centre for Invasion Biology, Department of Botany and Zoology, Stellenbosch University, Stellenbosch, South Africa; University of Minnesota

**Keywords:** bacterial dynamics, biochemistry, microbiome, sediment, fiddler crabs, microtidal, extreme environment, arid mangrove

## Abstract

In intertidal systems, the type and role of interactions among sediment microorganisms, animals, plants and abiotic factors are complex and not well understood. Such interactions are known to promote nutrient provision and cycling, and their dynamics and relationships may be of particular importance in arid microtidal systems characterized by minimal nutrient input. Focusing on an arid mangrove ecosystem on the central Red Sea coast, we investigated the effect of crab bioturbation intensity (comparing natural and manipulated high levels of bioturbation intensity) on biogeochemistry and bacterial communities of mangrove sediments, and on growth performance of *Avicennia marina*, over a period of 16 months. Along with pronounced seasonal patterns with harsh summer conditions, in which high sediment salinity, sulfate and temperature, and absence of tidal flooding occur, sediment bacterial diversity and composition, sediment physicochemical conditions, and plant performance were significantly affected by crab bioturbation intensity. For instance, bioturbation intensity influenced components of nitrogen, carbon, and phosphate cycling, bacterial relative abundance (i.e., *Bacteroidia, Proteobacteria* and *Rhodothermi*) and their predicted functionality (i.e., chemoheterotrophy), likely resulting from enhanced metabolic activity of aerobic bacteria. The complex interactions among bacteria, animals, and sediment chemistry in this arid mangrove positively impact plant growth. We show that a comprehensive approach targeting multiple biological levels provides useful information on the ecological status of mangrove forests.

**IMPORTANCE** Bioturbation is one of the most important processes that governs sediment biocenosis in intertidal systems. By facilitating oxygen penetration into anoxic layers, bioturbation alters the overall sediment biogeochemistry. Here, we investigate how high crab bioturbation intensity modifies the mangrove sediment bacterial community, which is the second largest component of mangrove sediment biomass and plays a significant role in major biogeochemical processes. We show that the increase in crab bioturbation intensity, by ameliorating the anoxic condition of mangrove sediment and promoting sediment bacterial diversity in favor of a beneficial bacterial microbiome, improves mangrove tree growth in arid environments. These findings have significant implications because they show how crabs, by farming the mangrove sediment, can enhance the overall capacity of the system to sustain mangrove growth, fighting climate change.

## INTRODUCTION

Sediment microbial dynamics in the intertidal zone are affected by fluctuations in key environmental factors (1–3), compounding out-of-phase diel and tidal cycles, along with other sources of variability. These fluctuations affect sediment temperature, salinity, and organic matter availability (4), resulting in changing microbial community diversity, evenness, and composition in saltmarshes (5), seagrass beds (6) and mangroves (7, 8). In addition to environmental variability, biological processes also influence sediment microbial community dynamics in intertidal sediments. Animal-mediated bioturbation is a prevalent process which introduces spatial environmental heterogeneity by altering the physical and biochemical sediment environment (9–11). Bioturbation enhances oxygen penetration into otherwise typically waterlogged and anoxic intertidal sediments on a millimeter scale (11). Consequently, there are significant changes in redox conditions which, in turn, shape the sediment microbiome by triggering a cascade of effects which shifts the predominant microbial respiratory pathways from anoxic to oxic ones (12–16).

Recently, Booth et al. (15) reported the so-called ‘halo effect’ created by fiddler crabs around their burrows, involving higher redox potential and modified microbial community composition and structure on a small spatial scale in bioturbated areas compared to that in unbioturbated areas. By altering the redox state in sediments, burrowers can potentially enhance or suppress certain microbial metabolisms, such as minimizing the activity of methanogenic archaea (17). Furthermore, burrowing animals, by modifying the overall physicochemical conditions of the sediments, drive the selection of different microbiomes in different burrow compartments compared to the surrounding sediments (18).

The interaction between bioturbators and the sediment microbiome, and the associated effects on nutrient cycling, also plays an important role in intertidal vegetated arid systems due to their oligotrophic conditions (19). Qashqari et al. (20) recently proposed that high crab density may reduce the cyanobacterial biofilm on the surface of mangrove sediment in an arid mangrove in the Red Sea, causing a decrease in nitrogen fixation rate and therefore in the level of nitrogen input in the system, which could affect plant growth. However, how the interaction between microorganisms, animals, and sediment influences plant fitness and homeostasis is still overlooked.

Here, we studied a microtidal arid mangrove system on the central Red Sea coast. Arid mangroves are characterized by almost zero rainfall and a lack of riverine input, and have reduced supplies of allochthonous carbon, nutrients, and organic matter (20–26). Environmental variability in these systems is therefore minimal (with the exception of seasonal factors), rendering them useful models for studying the interactions among components of mangrove ecosystems (i.e., animals, sediment microbes, and the sediment environment) and their potential impacts on plant growth. This study aims to identify the effect of bioturbation intensity on sediment physicochemical conditions, bacterial community assembly, and plant growth by testing the hypotheses that increasing bioturbation intensity (i.e., a high number of crabs) (i) affects mangrove sediment physicochemical conditions (biogeochemistry) and associated bacterial communities (alpha and beta diversity) consistently across seasons, and (ii) improves mangrove plant performance in a mature *Avicennia marina* stand.

## RESULTS

### Sediment environment.

**FIG 1:**
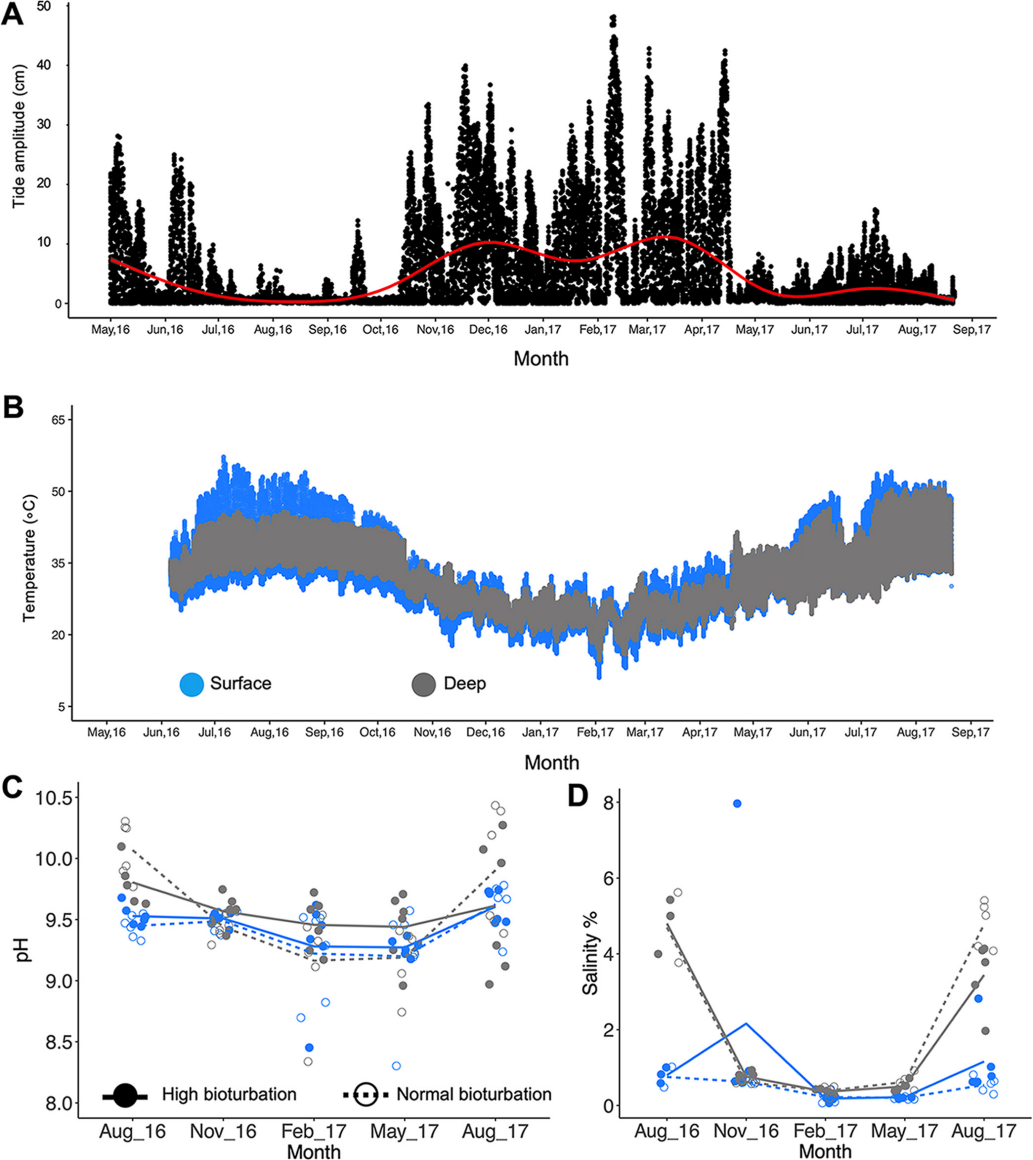
Environmental variability at the Central Red Sea Saudi Arabia mangrove study site from May 2016 to August 2017 for deep and surface sediment. (A) Overall tidal amplitude; red line represents the locally estimated scatterplot smoothing regression to summarize the tidal inundation level across the year; (B) sediment temperature, (C) pH, and (D) salinity in surface and deep sediment. Data were not retrieved for May 2016.

In the mangrove ecosystem studied, tidal amplitude ranged between 0 and 50 cm and was lowest in the summer months (Fig. 1A). Sediment surface temperature was highest from July to August, peaking at 58°C, while it dropped to a range of 8 to 10°C in the deep sediments (Fig. 1B). Alkaline and hypersaline conditions prevailed in surface sediments (Fig. 1C and D), but deep sediments were buffered with similar values throughout the year. Both sediment pH (manylm, *P *<* *0.001; Table S1A in the supplemental material) and sediment salinity (manylm, *P *<* *0.001; Table S1B) significantly varied according with the interaction of ‘depth,’ ‘month,’ and ‘bioturbation intensity’. Notably, higher bioturbation intensity resulted in a lower pH in the winter season, while the reverse was true in the dry season. Salinity was lower in the normal bioturbated surface sediment in November 2016 and August 2017, but did not differ in the deeper sediment, except for lower salinity in the highly bioturbated sediment in August 2017 only (Fig. 1C and D). Biogeochemical sediment signature was significantly affected by interactions between ‘month,’ ‘depth,’ and ‘bioturbation intensity’ (manylm, *P *<* *0.01; Table S1C, Fig. S3). Overall, particulate organic carbon (POC) and particulate organic nitrogen (PON) levels were higher in February, May, and August 2017 and particularly high in the surface of normal bioturbation intensity sediments compared to those subjected to high bioturbation intensity (Fig. S3A and B). Conversely, particulate inorganic carbon (PIC) was higher in May and November 2016 compared to that in the rest of the months, in both deep and subsurface high and normal bioturbation intensity sediments (Fig. S3C). Particulate inorganic nitrogen (PIN) was higher in surface sediments, particularly in highly bioturbated sediments, while it was lower in the subsurface and deep layers both in high and normal bioturbated sediments (Fig. S3D). Phosphate was significantly higher in surface sediments exposed to normal bioturbation intensity (Fig. S3E). Nitrate and nitrite showed distinct patterns related to seasons, both being more abundant in the colder months (as was PON). Nitrite in surface sediments was observed to be lower during high bioturbation intensity in May 2017 (Fig. S3F and G). Sulphate in surface sediments was highest in the hottest months (August 2016 and August 2017; Fig. S3H). Silicate concentration was higher overall during the colder months and was generally modulated by crab bioturbation intensity (Fig. S3I). Sediment pH was alkaline during the hottest and driest period and more acidic in the highly bioturbated sediments (Fig. S3L).

### Bacterial community dynamics.

**FIG 2:**
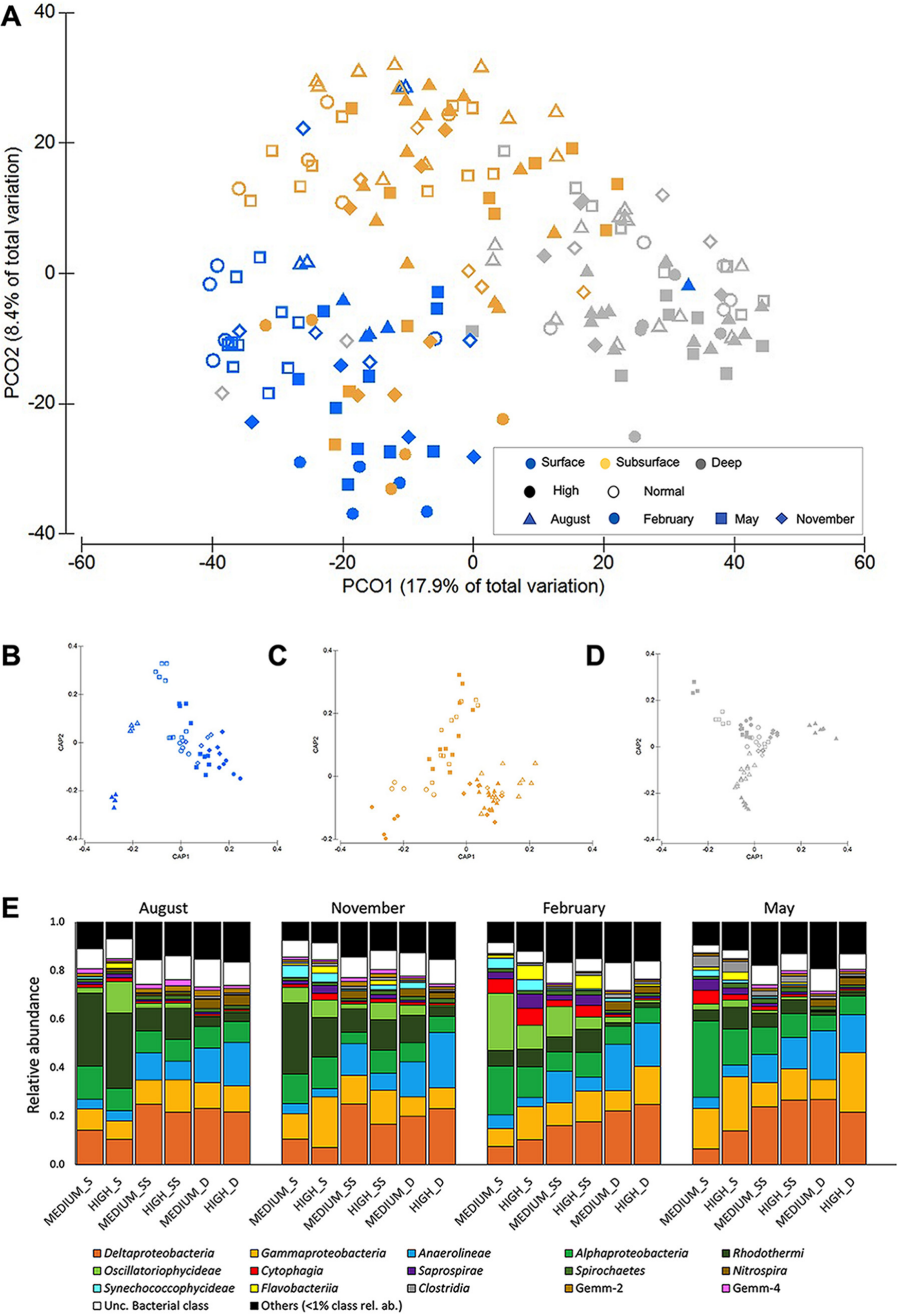
(A) Principal coordinate analysis (PCoA) of bacterial community composition across different bioturbation intensities, depths, and months of sampling. (B to D) Canonical analysis of principal coordinates (CAP) ordination to clarify the pattern of community change in each surface sampled: surface (B, blue), subsurface (C, yellow), and deep (D, gray). (E) Overall taxonomic composition of the bacterial community at the three sampling depths (S: surface, 0 to 0.5 cm deep; SS: subsurface, 0.5 to 1.0 cm; D: deep, 5 to 5.5 cm) across sampling times at two different levels of bioturbation intensity (normal and high-intensity bioturbation).

Bacterial alpha diversity, measured by both species richness (number of operational taxonomic units [OTUs]) and Shannon index, changed significantly according to the interactions of ‘month,’ ‘depth,’ and ‘bioturbation intensity’ (manylm, *P *<* *0.05; Table S2A,B; Fig. S4A,B). Quantitative PCR analysis also detected significantly different abundances of bacteria (measured as the number of bacterial 16S rRNA gene copies per gram of sediment) at different ‘months,’ ‘depths,’ and ‘bioturbation intensities’ (manylm, LR [likelihood ratio statistic]_5,36_ = 5.61, *P *<* *0.001; Fig. S4C). Likewise, bacterial community composition changed significantly throughout the sampling period, at different depths and with changing bioturbation intensity (mayglm, *P *<* *0.01 in all cases; Table S2C; Fig. 2A to E).

Bacterial communities were dominated (on average) by *Alphaproteobacteria* (~15%), *Gammaproteobacteria* (~10%), Deltaproteobacteria (~15%), *Anaerolinae* (~10%), *Rhodothermi* (~5%), and *Oscillatoriophycideae*. Most bacterial classes were differentially distributed across the treatments, with *Rhodorthermi* enriched in the surface sediment during the warm period and Deltaproteobacteria in deep sediment. The observed changes in bacterial community composition were mainly driven by salinity and PON (DistLM, Akaike information criterion [AICc] = 657.4, *R*^2^ = 0.14).

**FIG 3:**
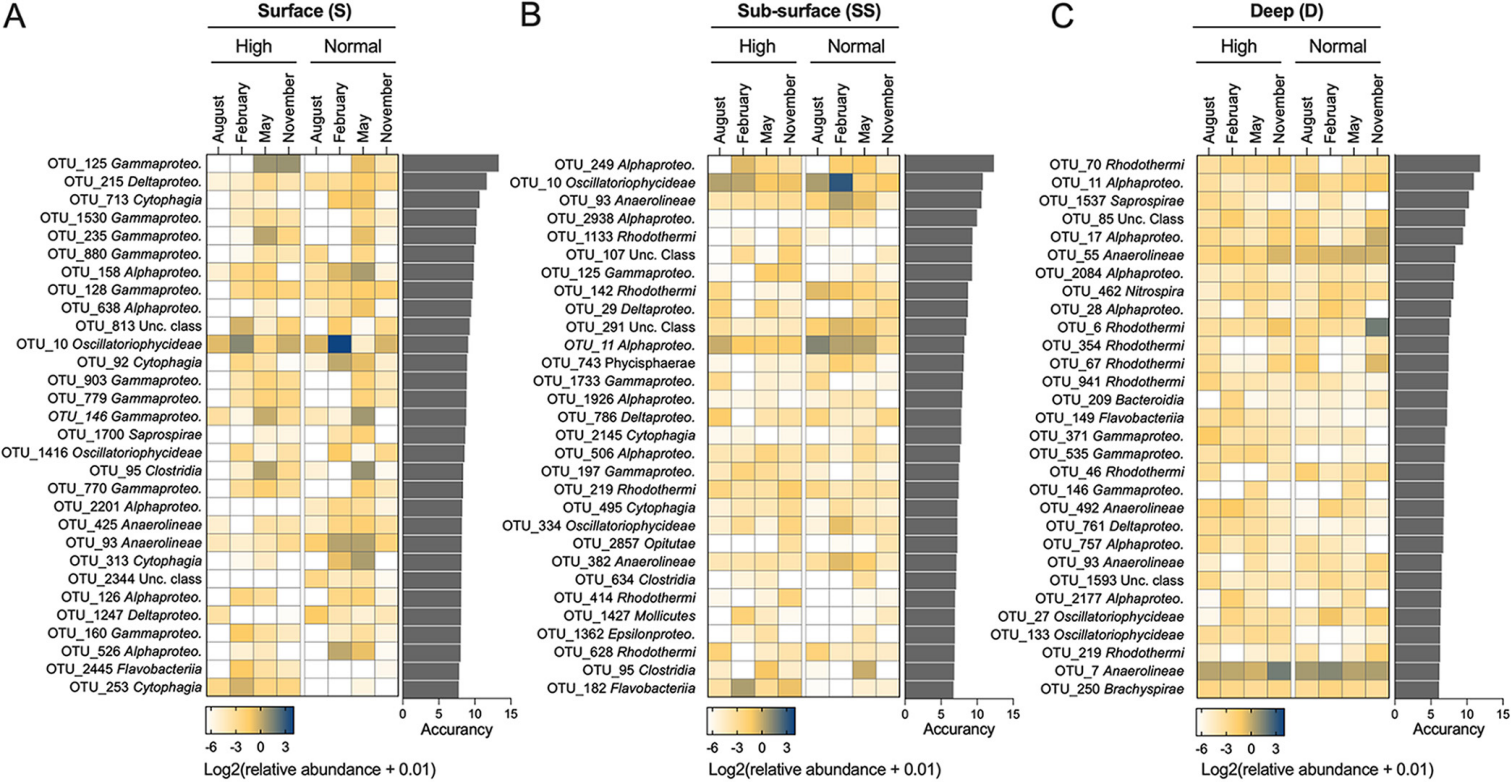
Top 30 identity operational taxonomic units (OTUs) used to discriminate bacterial communities in surface, subsurface, and deep sediment at different levels of bioturbation intensity across the sampling season. The assigned taxonomy of each taxon is displayed at the class level. Bar plots show the importance values of each OTU, estimated by random forest model as Mean Decrease Accuracy (%IncMSE).

A random forest model was used to distinguish microbial OTUs which could discriminate microbial communities with different bioturbation intensities over the sampling period for the three different sediment depths (Fig. 3). Ranked by their importance value, the top 30 bacterial OTUs mainly belonged to *Gammaproteobacteria* and *Alphaproteobacteria*. The three depths shared only 1 discriminant OTU and had 25 surface and subsurface and 26 deep unique discriminant OTUs. At the surface, *Gammaproteobacteria*, *Alphaproteobacteria*, *Cytophagia*, and *Oscillatoriophycideae* were the most represented discriminant classes. In the subsurface and deep, *Rhodothermi* was the main discriminant class together with *Alphaproteobacteria*, but not *Gammaproteobacteria* (Fig. 3).

### Functional signatures of mangrove sediment bacterial communities.

Fluorescein diacetate analysis (FDA) hydrolysis rate, used as a proxy of microbial activity in sediments, significantly changed with the interaction of ‘depth,’ ‘month,’ and ‘bioturbation intensity’ (manylm, LR_10,179_ = 10.51, *P *<* *0.001; Fig. S3D). Overall, the FDA hydrolysis rate was significantly lower in surface sediments compared to that in subsurface and deep sediments at different bioturbation intensities across the sampling period. When predicted ecological functions were assigned to bacterial OTUs (Fig. S5), we recorded significant changes in community functionality according to the interactions of ‘bioturbation intensity × depth,’ ‘bioturbation intensity × month,’ and ‘month × depth,’ but not for their full interaction (Table S2D). Photoautotrophy was significantly higher in the surface sediments (Fig. S5A), with the highest levels in normal bioturbated sediment in May 2016, November 2016, and February 2017. Overall, a higher bacterial OTUs assignment to the function of sulfate respiration was recorded in subsurface and deep sediments (Fig. S5E), which accords with the higher concentration of sulfate recorded at such depths (Fig. S3G). This portion of the bacterial community was consistently more abundant in subsurface and deep sediments than in surface sediments and was least abundant in the summer (August 2016 and 2017). A higher number of bacterial OTUs performing sulfate respiration in deep sediments was detected with higher bioturbation intensity in the months of November 2016 and May 2017 (*P *<* *0.01). In May and August 2016, bioturbation intensity was positively associated with the number of bacterial OTUs assigned to the function of cellulolysis in deep sediment (*P *<* *0.05; Fig. S5B). In May 2017, the number of OTUs assigned to nitrification and nitrate reduction in the subsurface and deep layers increased with increasing bioturbation intensity (*P *<* *0.05; Fig. S5C and F). In surface sediments, the number of bacterial OTUs assigned to photoheterotrophic bacteria was positively associated with bioturbation intensity in all months except August 2016 and 2017 (Fig. S5G). No significant pattern was found for nitrogen fixation (Fig. S5D) except in the surface sediments, where it reached the highest values in the highly bioturbated sediments in November 2016 and August 2017 and in the normal bioturbated sediments in February 2017. Instead, sulfite respiration was predominantly higher in the subsurface and deep sediments (Fig. S5H).

### Plant growth and correlation between biotic and abiotic mangrove components.

**FIG 4:**
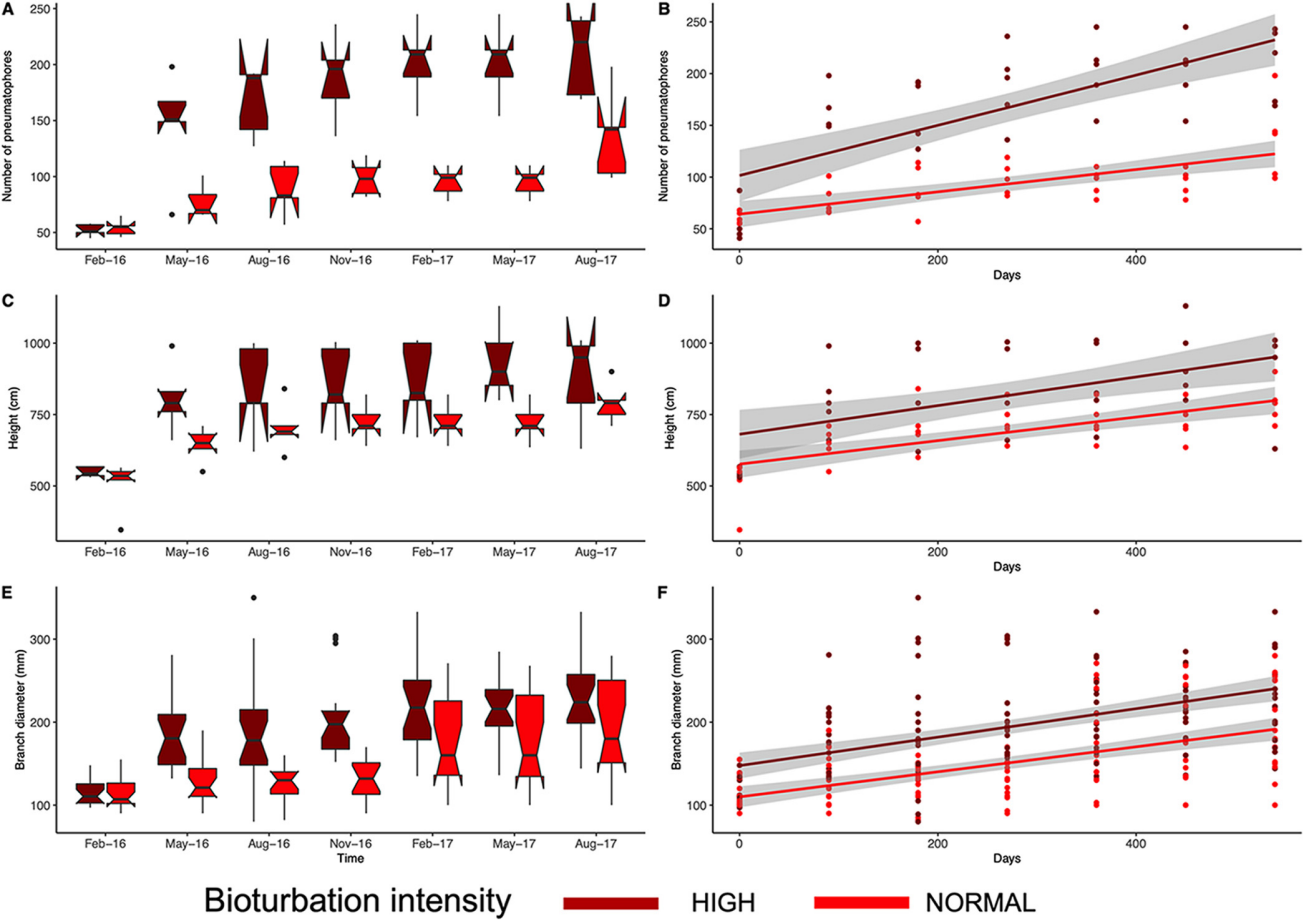
*Avicennia marina* performance during the survey period: (A and B) number of pneumatophores, (C and D) tree height, and (E and F) branch diameter are reported as indicators of plant growth and development in high and normal bioturbation intensity. The graphs in panels A, C, and E (boxplots and notches) describe the growth of the plants over the duration of the experiment at highly and normal bioturbation intensity. Graphs in panels B, D, and F show plant growth over the duration of the experiment, represented as a function of the days, with trendlines for high and normal bioturbation intensities.

**FIG 5:**
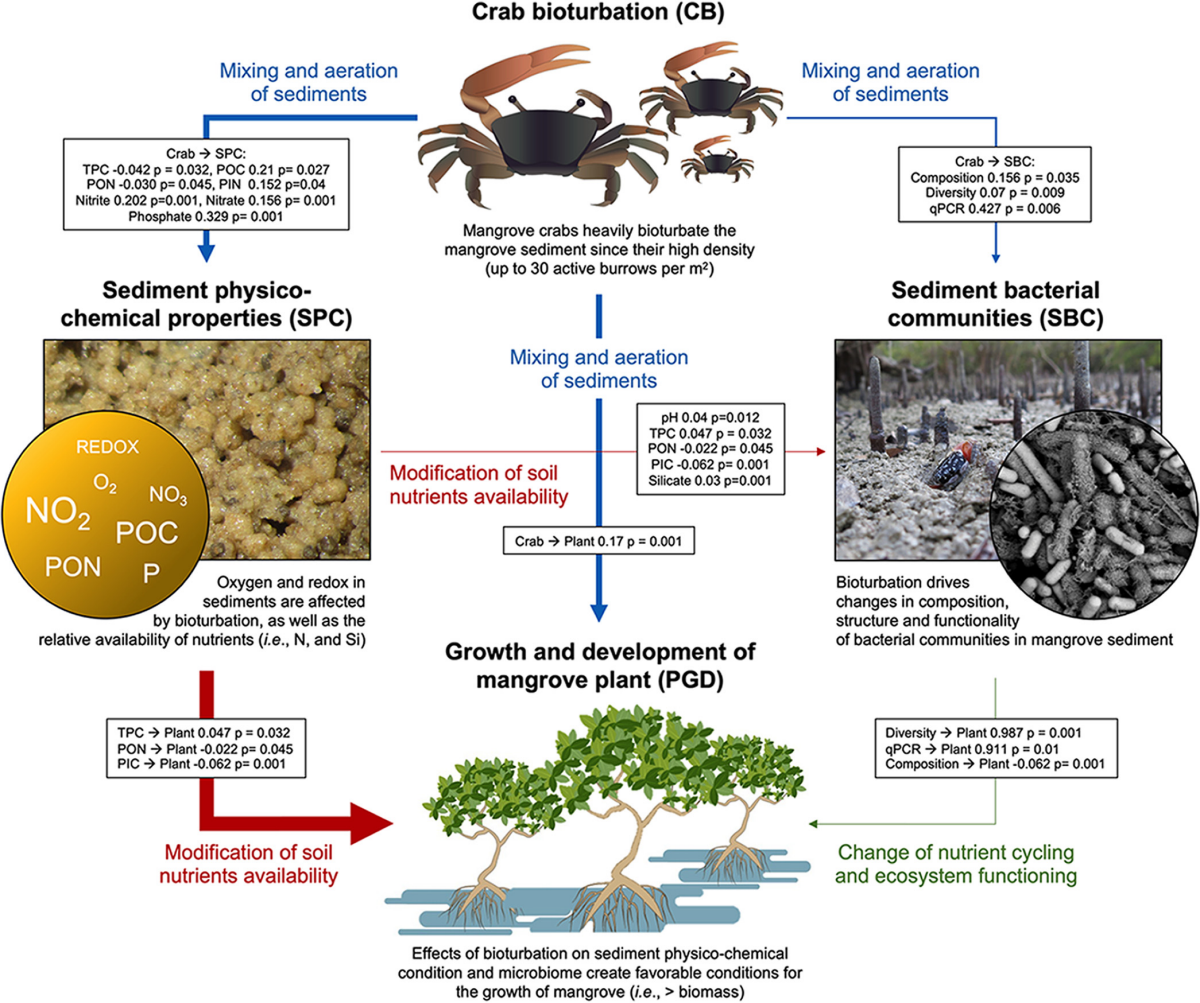
Structural equation model assessing the effects of the studied factors on plant performance. Numbers superimposed on the arrows in boxes reflect the strength of the effect of each variable. Only significant effects (*P* < 0.05) are shown. The model was satisfactorily fitted to the data, as suggested by the non-significant χ^2^ values, root mean square error of approximation value of 0.092, and CFI value of 0.89. The strength of correlations between bioturbation intensity and the different parameters studied are reflected in the different size of the arrows, which are proportional to the Mantel statistic R values based on the Pearson’s correlation. Mangrove ecosystems are densely colonized by burrowing fauna, which have a significant impact on sediment physicochemical conditions (SPC). In particular, TPC, particulate organic carbon (POC), particulate organic nitrogen (PON), particulate inorganic nitrogen (PIN), nitrate, nitrite, and phosphate were the main variables affected by the crabs. By aerating, mixing, and modifying the sediment structure, bioturbation affects the physicochemical properties of the soil and its sediment bacterial community (SBC), including the alpha and beta diversity and the quantity of 16S rRNA gene copies. These three variables also significantly affected plant growth. Among the sediment chemical parameters, TPC, PON, and particulate inorganic carbon (PIC) significantly affected plant growth. Ultimately, a significant effect of bioturbation intensity on the plant growth was revealed, showing that crab bioturbation (CB) sustained plant performance (PGD) in particularly stressful situations, such as those in arid environments.

We found a significant effect of ‘bioturbation intensity’ and ‘month’ on plant growth parameters (number of pneumatophores: bioturbation intensity F_1,48_ = 120.44, *P *<* *0.01; month F_5,48_ = 5.07, *P *<* *0.01 [Fig. 4A and B]; plant height: bioturbation intensity F_1,48_ = 23.25, *P *<* *0.01; month F_5,48_ = 2.4, *P *<* *0.05 [Fig. 4C and D]; branch diameter: bioturbation intensity F_1,200_ = 72.25, *P *<* *0.01; month F_5,200_ = 8.87, *P *<* *0.01 [Fig. 4E and F]). We found significant positive correlations of plant growth parameters with soil physicochemical properties and bioturbation intensity, followed by sediment bacterial community beta diversity (Fig. 5). Using a structural equation model (SEM), we further assessed the effects of bioturbation intensity, microbial community diversity and composition, and sediment biochemistry on plant growth. Our models explained a large portion of plant growth to be related to changes in bioturbation intensity. Among all the variables, total particulate carbon (TPC), PON, PIN, POC, nitrate, nitrite, and phosphate were affected by bioturbation intensity. pH, TPC, PON, PIC, and silicate had the strongest effects on bacterial richness and composition. Plant growth was affected most significantly by TPC, PON, and PIC. Similarly, bacterial community composition, richness, and abundance had significant effects on plant growth (Fig. 5).

## DISCUSSION

To investigate the effects of animal bioturbation intensity on microbial communities and plants in intertidal sediments, we studied an arid dwarf mangrove system on the Red Sea coast which was exposed to marked seasonality in temperature and salinity and limited precipitation (26–29). Although plant productivity is curbed in arid mangroves due to harsh environmental and oligotrophic conditions (24), these environments present good models for such a study because of their minimal external nutrient inputs from fluvial freshwater and rainfall compared to other tropical systems, which reduces ecological background noise. Overriding the seasonal patterns in Red Sea mangrove ecosystems, we show here that macrofaunal bioturbation intensity influenced sediment bacterial community composition, taxonomy, function, diversity and activity, sediment biogeochemistry, and plant growth performance (Fig. 5). The interplay of seasonal temperature variability and the density of active crab burrows, i.e., intensity of bioturbation (highest in cooler months), is complex, with a constant but variable effect on the sediment environment and bacterial community at different times of the year. The summer months (May and August 2017) were associated with reduced microbial activity in the surface and subsurface sediments, likely due to the seasonal depression of the Red Sea sea level, extreme temperature, and salt accumulation. For instance, the ambient air temperature was very high in the summer months (more than 45°C from June to August) and, correspondingly, sediment temperature was also high (more than 50°C) due to the low frequency, or absence, of flooding by seawater (microtidal regime). These environmental features were reflected in the hypersaline sediment conditions during the summer, with a salt crust forming on the mangrove sediment surface. These conditions are typical and cyclical in the Red Sea because the water from the basin flows into the Indian Ocean during the summer due to a low-pressure system. This cycle can be further exacerbated in El Niño years when sea level is reduced even further (30, 31).

In mangrove successions, vegetation substantially influences levels of organic matter and nutrients through their accumulation in the sediments (32), which drives the development of stratified layers of microorganisms (25). At our study site, a well-developed layer of cyanobacteria (photoautotrophic bacteria) was present on sediment surfaces that were not intensely affected by bioturbation, a major factor in determining the structure of these surfaces (33), together with an autochthonous organic litter layer. The latter is particularly important in mature mangrove stands in the Red Sea, where more than half of the accumulated organic matter in sediments originates from mangrove tissues (34). However, in arid systems, litterfall is typically low (35). While frequent tidal inundation significantly increases leaching from litter, in summer months, when tidal inundation diminishes in the Red Sea, this leaching is dramatically reduced.

Although crab grazing activity affects the total bacterial biomass in sediments, the seasonal variation of the microbial community was dictated by tidal cycles and sea level fluctuations. Remarkably, the same bacterial community composition was observed in the two consecutive study years (2016 and 2017) during the hottest period of the year (i.e., August), when the sea level is lowest and salinity highest. Nevertheless, sediment depth was also shown to be a major driver of microbial community composition due to the rapid shift to anoxic conditions with depth, in agreement with previous studies (15). In general, crab bioturbation intensity modulated the distribution of bacteria in the sediment. High bioturbation intensity mediates the presence of *Oscyllatoriophycidae* (increases) and *Rhodothermi* (decreases) in the surface sediments, of *Alphaproteobacteria* and *Anaerolinae* in the subsurface sediments, and of Deltaproteobacteria in the deep layers. The diversity of bacterial taxa promoted by bioturbation may be related to the increased number of niches created by the grazing, digging and sediment reworking by crabs (see supplementary video [14]). In the deep and subsurface sediments, physicochemical conditions can reduce the capability of bacteria to exploit the available niche due to strong selective pressures; for example, a sharp decrease in oxygen can affect the distribution of aerobic bacteria, such as members of *Gammaproteobacteria*.

In terms of predicted bacterial community functions, photoheterotrophs significantly increased during May and November, especially in surface sediment, while photoautotrophs were related to increasing bioturbation intensity in surface sediment during the cooler months (except November 2016). This relationship is likely explained by crab grazing on the sediment surface (15, 20), which can enrich some photoheterotrophic bacteria (i.e., *Alphaproteobacteria*) to the detriment of photoautotrophic bacteria (i.e., *Cyanobacteria* mats/biofilms); by contrast, the positive effect of increased bioturbation intensity on the relative abundance of *Cyanobacteria* may be linked to the fact that during the summer period, crab grazing can be reduced due to high temperature and salinity, as observed in other locations (36). The significant decrease in sulfate respiration in deep sediment with the increase in bioturbation intensity may be associated with decreased sulfate reduction due to the different redox conditions determined by the crab burrows, which also increase the level of nitrification (15). Bioturbation intensity was a driver of the environmental parameters salinity and PON, which in turn were the main environmental drivers responsible for structuring bacterial community composition, along with bioturbation intensity. Burrowing and sediment reworking activity is known to shift the dominant respiratory pathway in mangrove sediment, a sulfur-rich environment, away from sulfate reduction due to oxidation of reduced compounds (37). In this study, the effect of bioturbation on sediment sulfate concentration was variable. The number of bacteria assigned to the function of sulfate respiration in deep sediment increased with increasing bioturbation intensity during certain months, while a reduction in bioturbation intensity was associated with an overall increase in sulfate across all depths during the summer months. Although variable, this result suggests that sulfate accumulates in sediment with reduced bioturbation intensity. Periods of desiccation in mangroves have been shown to increase the diffusion of oxygen into sediment in semi-arid Brazilian mangroves, thereby increasing redox conditions and oxidation (38). Higher crab densities during drier periods were correlated with a high level of oxidation of reduced compounds (39).

The observation that nitrogen (inorganic and organic) concentrations were higher at cooler times of the year and lowest in the summer may be explained by the activity of photosynthetic cyanobacteria, which are temperature-dependent and do not fix nitrogen beyond 35°C, as previously shown by Qashqari et al. (20). Macrofaunal burrowing in intertidal sediment can enhance rates of closely coupled nitrification-denitrification in saltmarshes (40) and estuaries (41). Bioturbation intensity was positively associated with increases in nitrite and PIN during the cooler months. The negative effects of bioturbation intensity on sediment PON concentration and the relative abundance of bacteria performing nitrogen fixation in surface and subsurface sediment we observed are likely due to crab grazing on cyanobacteria. We propose that bioturbation enhances the presence of bacteria which make limiting factors in arid systems, such as iron and phosphorous, available (21), while flushing of the crab burrows during high tide may contribute to increasing nutrient and oxygen supply and reducing sulfide accumulation, which may favor mangrove growth. The link between crab activity and mangrove growth enhancement is likely to include a suite of both direct and indirect mechanisms.

Despite the complexity of the system studied, we detected significant correlations among plant parameters, sediment biogeochemistry, and bioturbation intensity (Fig. 5). Plant parameters were also positively correlated with bacterial community composition. Even though the ecological mechanisms are yet to be identified in future studies, this series of correlations evidences a significant relationship between the different ecosystem components investigated (Fig. 5). Mangrove ecosystems worldwide have a characteristic low nitrogen/carbon ratio and low phosphorus concentration (34, 42–44), but those in the central Red Sea are particularly oligotrophic, with acutely low concentrations of available phosphorus and iron in the sediment (21), leading to stunted trees (24). Recycling of nutrients should thus be particularly efficient in this system, with microbial activity being responsible for major nutrient transformations crucial for ecosystem functioning and plant productivity (45, 46). The observed positive correlation between bioturbation intensity and plant growth performance (height and branch diameter) of *A. marina* mangrove trees in this study suggests a beneficial relationship. Interestingly, the number of pneumatophores in bioturbated sediment correlated positively with bioturbation intensity. This implies that bioturbation intensity, by changing the sediment biochemistry, creates favorable conditions for pneumatophore proliferation due to the enhancement of nutrients. In oligotrophic habitats, proliferation of roots in nutrient-enhanced microsites contributes to overall nutrient conservation by plants (47). It has been shown that *A. germinans* roots proliferate in the channels left by decaying roots (47) and this could explain the increased number of pneumatophores with increased bioturbation intensity. Plant roots could exploit crab burrows to grow, therefore generating more pneumatophores. Another possible explanation is that intense bioturbation creates environmental conditions suitable for bacteria with plant-promoting effects that can enhance the growth of plant roots and pneumatophores (48). Our results corroborate earlier studies of the effects of crabs on plant productivity in intertidal regions, pointing out that bioturbation-induced changes in the microbial community, rather than only variation in sediment biogeochemical characteristics, are beneficial for plant performance (49).

Our 16-month experimental manipulation of an arid mangrove revealed a significant relationship between the biotic and abiotic components of this ecosystem, namely, crab bioturbators, the sediment bacterial community selected by the activity of these animals, sediment biogeochemistry, and plant growth. While we did not explore the mechanistic processes of these relationships here, our data show that the mangrove sediment microbiome is mediated by multi-faceted drivers ranging from environmental seasonality to the activity of bioturbating macrofauna, which, together, sustain plant performance. However, there are certainly other factors besides those studied here that could affect such multifaceted relationship.

Yet, despite the high seasonal variability that arid mangroves of the Red Sea experience, which clearly influences changes in microbial dynamics, sediment bacterial community composition structure also correlated positively with bioturbation intensity and mangrove plant growth, highlighting the intrinsic connections between microbes, animals, and plants. We therefore stress the important role of the ecosystem-engineered microbiome as an essential and frequently overlooked component that supports the overall ecosystem functionality of mangrove forests.

## MATERIALS AND METHODS

### Experimental design.

The study was conducted in an arid fringe *A. marina* mangrove stand in the central Red Sea, Saudi Arabia (Ibn Sina Field Research Station, Thuwal: 22°20′25.03″N, 39°5′17.41″E; Fig. S1A and B). Ten random 3 × 3 m experimental plots in an area with mature vegetation were established at the same level of tidal inundation; plots were delimitated using plastic pipes and orange ropes with a single mangrove tree (of similar height) per plot (Fig. S1C). To assess the impact of different intensities of crab bioturbation in each plot, we enriched half of the plots with higher densities of crabs belonging to the species *Cranuca inversa* (Ocypodidae) and *Dotilla sulcata* (Dotillidae) 3 months prior to the start of the survey, while the other half of the plots were maintained at a natural density, as described by Qashqari et al. (20). The enrichment approach was adopted for two reasons. First, it is not possible to effectively remove large numbers of crabs from sediment without altering the structure and therefore the microbiome, since it requires digging sediment to a depth of at least 20 cm. Secondly, crab enrichment is effective due to the high burrow fidelity of the studied animals, which tend to maintain the same burrow if the environmental conditions are favorable (15, 50, 51). The number of active burrows was counted in each sampling month to monitor bioturbation intensity (Fig. S2). For analytical purposes, bioturbation intensity was considered a categorial variable with two levels, ‘high’ and ‘normal,’ throughout the experimental period; bioturbation intensity within the enriched plots was significantly higher than that in the non-enriched plots (analysis of variance: F_1,97_ = 11.59; *P *<* *0.005).

### Environmental monitoring and sampling.

To continuously record sediment temperature in the mangrove, six Onset HOBO U22-001 loggers were placed in the sediment at the surface and at a 15-cm depth. Two Onset HOBO U20L-01 loggers were set up to record the water level. Since the plots were set up at the same tidal level, we deployed the sensors randomly among the plots to be able to describe the overall environmental characteristics of the area. Field sampling was carried out every 3 months spanning a 16-month period (May, August, and November 2016; February, May, and August 2017). Sediment was collected for environmental and microbial community analysis from the surface (0- to 0.5-cm depth), subsurface (0.5- to 1.0-cm depth), and deep layers (5- to 5.5-cm depth) of each plot. For each plot, sediment was collected using a sterile spatula from an area of approximately 5 cm^2^. For each sample depth in each plot, we mixed sediments from three randomly selected points after collection in a 50-mL Falcon tube using a spatula. Samples were stored on ice in the field and transported back to the lab within 1 h of sampling. From each sample, 1 g of sediment for fluorescein diacetate analysis was immediately processed to assess microbial activity, 5 g was used to measure salinity and pH, 0.5 g was frozen at −20°C for subsequent DNA extraction, and 10 g was frozen at −20°C for biogeochemical analysis.

At each sampling time, plant growth was monitored by measuring the height of the tree inside each plot, the diameter of each branch of that tree at 10 cm from the ground (subsequently calculating mean branch diameter), and the total number of pneumatophores in each plot.

### Physicochemical and microbial analyses of sediments.

Sediment salinity was measured by combining sediment and distilled water (1:5 ratio; 5 g soil, 25 mL water) before mixing on a rotary shaker for 30 min; the salinity of the solution was recorded with a hand-held refractometer once it had settled (52). Biogeochemical sediment analysis was performed at GEOMAR (Kiel, Germany). Total particulate carbon and nitrogen were analyzed (Thermo Finnegan Flash EA1112) and measurements of POC and PON were obtained using an acidification method (53). PIC and PIN measurements were attained after subtraction of the organic component from the total. Nutrient analyses (nitrate, nitrite, silicate, and phosphate) were performed in deionized water leaches (54) using a standard auto-analyzer (Seal AA3) following the protocols of Grasshoff et al. (55). Sulphate concentrations were obtained using ion chromatography (56).

DNA was extracted from 0.4 ± 0.05 g of each sediment sample using a MoBio Power Soil DNA extraction kit (MoBio, Jefferson City, MO). Using the primers 341F and 785R, the V3 to V4 hypervariable region of the 16S rRNA gene was amplified (following the methods of Callahan et al. [57]). A 96 Nextera XT Index Kit (Illumina) was used to prepare amplicon libraries, which were sequenced with pair-end sequencing in the BioScience Core Lab (KAUST, Thuwal, Saudi Arabia) using the Illumina MiSeq platform. Primers were trimmed from raw forward and reverse reads using Cutadapt (58), and the DADA2 pipeline (R software) was used to join and denoise merged reads (57). We used the SILVA v138 database (59) to assign taxonomy to sequence variants (SVs), obtaining 12,854 bacterial SVs (hereafter referred to as OTUs). The FAPROTAX database was used to assign bacterial OTUs to known metabolic or ecological functions (http://www.zoology.ubc.ca/louca/FAPROTAX [60]); 2,378 of 9,478 OTUs (25.1%) were assigned to at least one functional group, while the remaining 7,100 OTUs (74.9%) could not be assigned to any group (leftovers).

Copies of the bacterial 16S rRNA gene were quantified using the PCR protocol described below and the primer pair Eub338-Eub518 (61). Quantitative PCRs were performed in a Rotor-Gene Q thermocycler (Qiagen, Hilden, Germany) using the GoTaq qPCR SYBR Green Master Mix (Promega, Madison, WI). DNA were first quantified using the Qubit dsDNA BR assay kit for each sample, then diluted to 2 ng/μL to be used as template DNA. PCRs were performed in a volume of 15 μL containing 1× GoTaq Master Mix (with 2 mM MgCl_2_), 100 nM of each primer, and 1.5 μL of template DNA. Quantitative PCR conditions for bacteria were 95°C for 2 min, 45 cycles at 95°C for 15 s, 53°C for 20 s, and 60°C for 20 s. At the end of the run, denaturation curves were obtained by performing re-naturation at 50°C for 180 s, followed by gradual denaturation for 91 cycles from 50°C to 95°C with an increase of 0.5°C/cycle every 5 s. Standard curves were created for each quantitative PCR (qPCR) assay, with a series of dilutions ranging from 50 to 5 × 10^7^ copies/μL. All standards and samples were run in triplicates. Concentration, in terms of copies/μL, for every sample was calculated from the standard curve. In all the qPCR assay runs, *R*^2^ varied between 0.99309 and 0.99908 and amplification efficiencies varied between 85% and 100%. Copy-numbers were corrected by the dilution factor. The standard curve was obtained by plotting the concentration values of the standards against the threshold cycle (*C_T_*) values calculated by the qPCR machine.

Microbial hydrolyzing activity was assessed by performing an FDA hydrolysis assay on each sediment sample. Specifically, within 1 h of sample collection, 1 g of sediment was added to 50 mL of sterile sodium phosphate buffer (60 mM [pH 7.6]). After dissolving 7.2 mM FDA substrate in acetone, 0.5 mL was added to each sample and incubated for 3 h in the dark on a rotary shaker set at 150 rpm. As a control, the same assay was performed on sterile (autoclaved) sediment. After 3 h, 2 mL of acetone was added to each sample to terminate the reaction. Using a spectrophotometer set at 490 nm, fluorescence was read and fluorescein concentration was calculated with reference to a previously calculated standard curve.

### Statistical analysis.

All statistical analyses were performed with R (62), GraphPad/PRISM (63), and PRIMER (64). Our experimental design incorporated three categorical orthogonal factors: ‘month’ (6 levels: May 2016, August 2016, November 2016, February 2017, May 2017, and August 2017), ‘depth’ (3 levels: surface, subsurface and deep), and ‘bioturbation intensity’ (2 levels: ‘normal’ and ‘high’). In all the statistical analyses, we considered the identity of the plot by controlling possible spatial and temporal pseudo-replications. Using the ‘*manylm()*’ function from the mvabund package (65) in R, we performed a 3-way analysis of variance to test the effects of ‘month,’ ‘depth,’ and ‘bioturbation intensity’ on pH, salinity, and sediment biochemistry. Data were plotted using boxplots with notches to visualize differences among factor levels; no overlapping notches indicate significant statistical differences among the boxplots.

A multivariate generalized linear model was used to test the effects of ‘month,’ ‘depth,’ and ‘bioturbation intensity’ on the bacterial community beta diversity, predicted functional assignment, alpha diversity measured as richness and Shannon index, the number of copies of bacterial 16S rRNA genes, and sediment FDA concentration using the R package mvabund (65). In both the manylm and mayglm functions, we included the offset argument set to the number of sequencing reads for each sample (66). After a centered log transformation using the function ‘*clr()*’ of the R package composition as specified previously (66), Principal coordinate analysis (PCoA) and canonical analysis of principal coordinates (CAP) was applied to explore variations in bacterial community composition across ‘depth,’ ‘month,’ and ‘bioturbation intensity’. Prior to running the above-mentioned statistical analyses, data were checked for normality and homogeneity of dispersion and transformed accordingly.

The number of total replicates varies with the success of DNA extraction; for example, in August 2016, DNA extraction was problematic due to elevated sediment salinity.

To find the best discriminant microbial OTUs among the sediment ‘bioturbation intensity’ and ‘depth’ across ‘month,’ classification random forest analysis was applied using the R package randomForest (67, 68) and evaluated by %IncMSE (Mean Decrease Accuracy), which describes the prediction ability of mean square error with randomly permuted variables. To determine which of the biochemical variables explained differences in bacterial community composition, we used distance-based multivariate analysis for a linear model in Primer (DistLM) (64), with significance provided by the corrected Akaike information criterion (AICc) (69). Prior to analysis, we tested for multi-collinearity using the non-parametric Spearman correlation and Draftsman’s plots in Primer on normalized data to ensure that the correlation coefficients were <0.85.

A Mantel test using Pearson’s correlation was used to study the relationships between sediment bacterial community composition (Bray-Curtis similarity matrix), sediment biochemistry, bacterial 16S rRNA gene copies, bioturbation intensity, and plant growth parameters (each data set as a Euclidean similarity matrix). The correlations were performed using vegan package 3.06 (70) and considered significant when *P* was * *<0.05 by using 999 permutations. We used a structural equation model to confirm the correlative result from the Mantel test on the effects of bioturbation intensity, soil biochemistry, bacterial diversity, and composition on plant performance using the package lavaan (71). All the data were normalized prior to modeling. The requirements of the parameters to fit the model included a root mean squared error of approximation of (RMSEA) <0.05, a low chi-square value (χ^2^), and a comparative fit index (CFI) > 0.85 criterion.

### Data availability.

The data set generated during this study is available in the NCBI SRA repository under the BioProject ID PRJNA813530 and at www.doi.org/10.11922/sciencedb.j00076.00097. The R scripts used in this manuscript are available in the Github community repository at https://github.com/MarcoFusi1980/MangrovePlot.
