# Supplementary material for: Bioturbation Intensity Modifies the Sediment Microbiome and Biochemistry and Supports Plant Growth in an Arid Mangrove System
Source: Microbiol Spectr. 2022 Jun 1;10(3):e01117-22. doi: 10.1128/spectrum.01117-22 (PMC9241789; doi:10.1128/spectrum.01117-22)

Supplemental Material For Publication

Bioturbation intensity modifies the sediment microbiome and biochemistry and supports plant growth in an arid mangrove system

Marco Fusi[1,2,†,*], Jenny Marie Booth[1,3,†], Ramona Marasco[1], Giuseppe Merlino[1], Neus Garcias-Bonet[1,4], Alan Barozzi[1], Elisa Garuglieri[1], Tumeka Mbobo[5,6,7], Karen Diele[2], Carlos M. Duarte[1,4], Daniele Daffonchio[1,*]

[1]Division of Biological and Environmental Science and Engineering (BESE), Red Sea Research Center (RSRC), King Abdullah University of Science and Technology (KAUST), Thuwal 23955-6900, Saudi Arabia

[2]School of Applied Sciences, Edinburgh Napier University, Edinburgh, UK

[3]Coastal Research Group, Department of Zoology and Entomology, Rhodes University, PO Box 94, Grahamstown 6140, South Africa

[4]Computational Bioscience Research Center (CBRC), King Abdullah University of Science and Technology (KAUST), Thuwal 23955-6900, Saudi Arabia

[5]National Research Foundation-South African Institute for Aquatic Biodiversity Institute, Makhanda, 6140, South Africa

[6]South African National Biodiversity Institute, Kirstenbosch Research Centre, Cape Town, South Africa

[7]Centre for Invasion Biology, Department of Botany and Zoology, Stellenbosch University, Stellenbosch, South Africa

[†]These authors contributed equally to this work.

*Correspondence to:

Email: marco.fusi@kaust.edu.sa; phone: +44(0)7342178812

Email: daniele.daffonchio@kaust.edu.sa; phone: +966 (0)12 8082884

Supplementary tables

Supplementary Table S1. Anova table of the analysis of covariance for sediment pH (**A**), salinity (**B**) and biochemistry (**C**). Df: degree of freedom; Res.df.: Residual degree of freedom; F: F-statistic; *P*: p statistic. Statistically significant terms are in bold.

A) pH	Df	Res.df.	F	*P*
Depth	1	142	4.63	**<0.001**
Month	5	137	2.97	**<0.001**
Bioturbation intensity	1	136	1.05	**<0.001**
Depth × Month	5	131	0.59	**<0.001**
Depth × Bioturbation intensity	1	130	2.51	**<0.001**
Month × Bioturbation intensity	5	125	0.95	**<0.001**
Month × Depth × Bioturbation intensity	5	120	1.45	**<0.001**

B) Salinity	Df	Res.df.	F	*P*
Depth	1	88	11.38	**<0.001**
Month	4	84	13.86	**<0.001**
Bioturbation intensity	1	83	0.13	**<0.001**
Depth × Month	4	79	20.33	**<0.001**
Depth × Bioturbation intensity	1	78	4.16	**<0.001**
Month × Bioturbation intensity	4	74	1.27	**<0.001**
Month × Depth × Bioturbation intensity	4	70	1.26	**<0.001**

C) Biochemistry	Df	Res.df.	F	*P*
Depth	2	105	57.56	**<0.005**
Month	5	100	46.29	**<0.005**
Bioturbation intensity	1	99	80.42	**<0.005**
Depth × Month	10	89	15.42	**<0.005**
Depth × Bioturbation intensity	2	87	59.38	**<0.005**
Month × Bioturbation intensity	5	82	16.36	**<0.005**
Depth × Month × Bioturbation intensity	10	72	23.09	**<0.005**

Supplementary Table S2. (**A**) Anova table of the analysis of variance for the sediment bacterial diversity (expressed as Shannon diversity) and (**B**) richness. Df: degree of freedom; Res.df.: Residual degree of freedom; LR: likelihood ratio statistic; *P*: p statistic. Statistically significant terms are in bold. (**C**) Anova table of the multivariate generalised linear model analysis of variance for bacterial community composition. Df: degree of freedom; R^2: proportion of the variance for the explanatory variable explained by the multivariate response variables, Deviance: Deviance explained; *P*: p statistic. Statistically significant terms are in bold. (**D**) Anova table of the multivariate analysis of variance of the predicted functions from the bacterial community. Df: degree of freedom; R^2: variance explained by each single terms; F: F statistic; *P*: *p* statistic

**A**) Richness	Df	Res.df.	LR	*P*
Bioturbation intensity	1	156	1.76	**0.001**
Depth	2	154	3.67	**0.001**
Month	5	149	10.53	**0.001**
Bioturbation intensity × Depth	2	147	1.93	**0.001**
Bioturbation intensity × Month	5	142	1.46	**0.001**
Depth × Month	10	132	1.16	**0.001**
Bioturbation intensity × Depth × Month	10	124	0.732	**0.001**

**B**) Shannon diversity	Df	Res.df	LR	*P*
Bioturbation intensity	1	157	1.95	**0.001**
Depth	2	156	0.53	**0.001**
Month	5	149	2.73	**0.001**
Bioturbation intensity × Depth	2	147	0.88	**0.001**
Bioturbation intensity × Month	5	142	1.82	**0.001**
Depth × Month	10	132	1.26	**0.001**
Bioturbation intensity × Depth × Month	10	124	1.32	**0.001**

**C)** Bacterial community composition	Df	R^2	Deviance	*P*
Depth	2	0.12	820.4	**0.001**
Month	5	0.09	439.5	**0.001**
Bioturbation intensity	1	0.03	133.9	**0.001**
Depth × Month	9	0.07	527.8	**0.001**
Depth × Bioturbation intensity	2	0.02	210.0	**0.006**
Month × Bioturbation intensity	5	0.03	234.9	**0.001**
Depth × Month × Bioturbation intensity	9	0.04	448.8	**0.029**
Residuals	143	0.54		

**D**) Function	Df	R^2	F	*P*
Bioturbation intensity	1	0.03	9.71	**0.001**
Depth	2	0.13	18.22	**0.001**
Month	5	0.13	7.23	**0.001**
Bioturbation intensity × Depth	2	0.02	2.11	**0.025**
Bioturbation intensity × Month	5	0.04	2.2	**0.001**
Depth × Month	9	0.07	2.13	**0.001**
Bioturbation intensity × Depth × Month	9	0.03	0.9	0.54
Residual	140	0.53		

Supplementary Figures

Supplementary Figure S1. (**A**) Study area location (central Red Sea) and its magnification (**B**). (**C**) Study site: a plot within the dwarf arid mangrove.

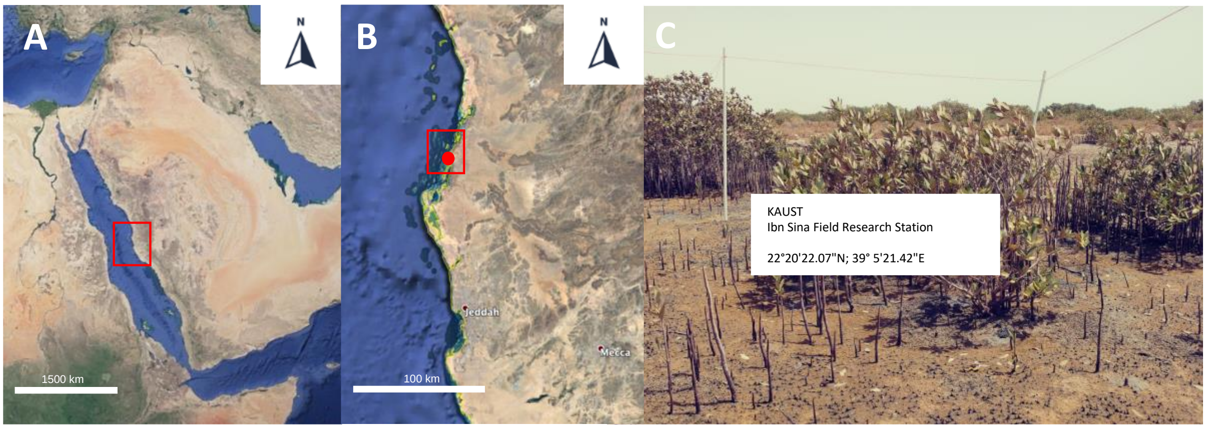

Supplementary Figure S2. Number of active crab burrows per square meter over the sampling period in plots with high and normal levels of bioturbation intensity.

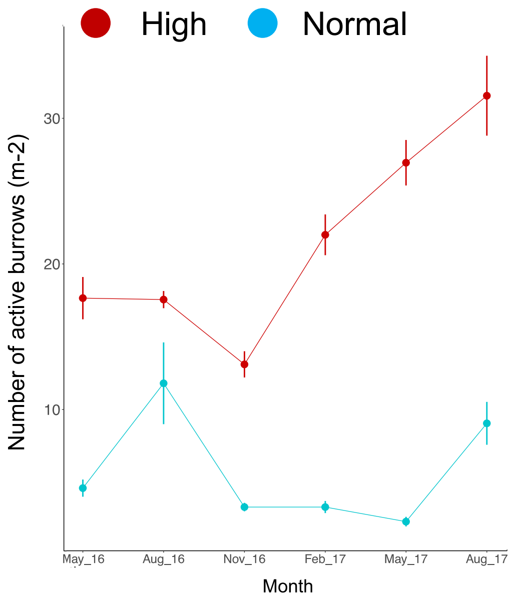

Supplementary Figure S3. Analysis of the (**A**) POC = particulate organic carbon; (**B**) PON = particulate organic nitrogen; (**C**) PIC = particulate inorganic carbon; (**D**) PIN = particulate inorganic nitrogen, (**E**) phosphate; (**F**) nitrite; (**G**) sulphate; (**H**) nitrate; and (**I**) silicate in relation to 'bioturbation intensity' and 'month'.

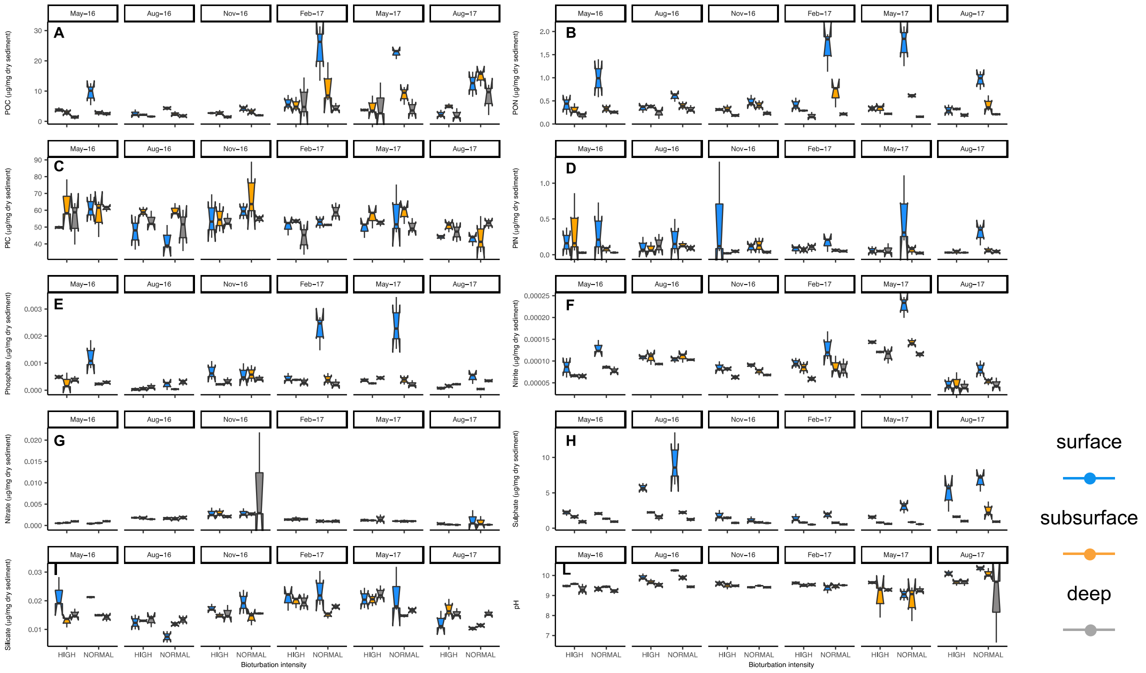

Supplementary Figure S4. Analysis of (A) bacterial alpha-diversity calculated as Shannon diversity and (B) bacterial richness, in relation to 'bioturbation intensity', 'depth' and 'month'. (C) Analysis of the bacterial 16S rRNA gene copies in relation to 'bioturbation intensity', 'depth' and 'month'. (D) Analysis of sediment microbial bacterial aerobic activity (FDA) in relation to 'bioturbation intensity', 'depth' and 'month'. May 2016 data were not collected and August 2016 data were biased by the high salinity of the sediment and therefore removed.

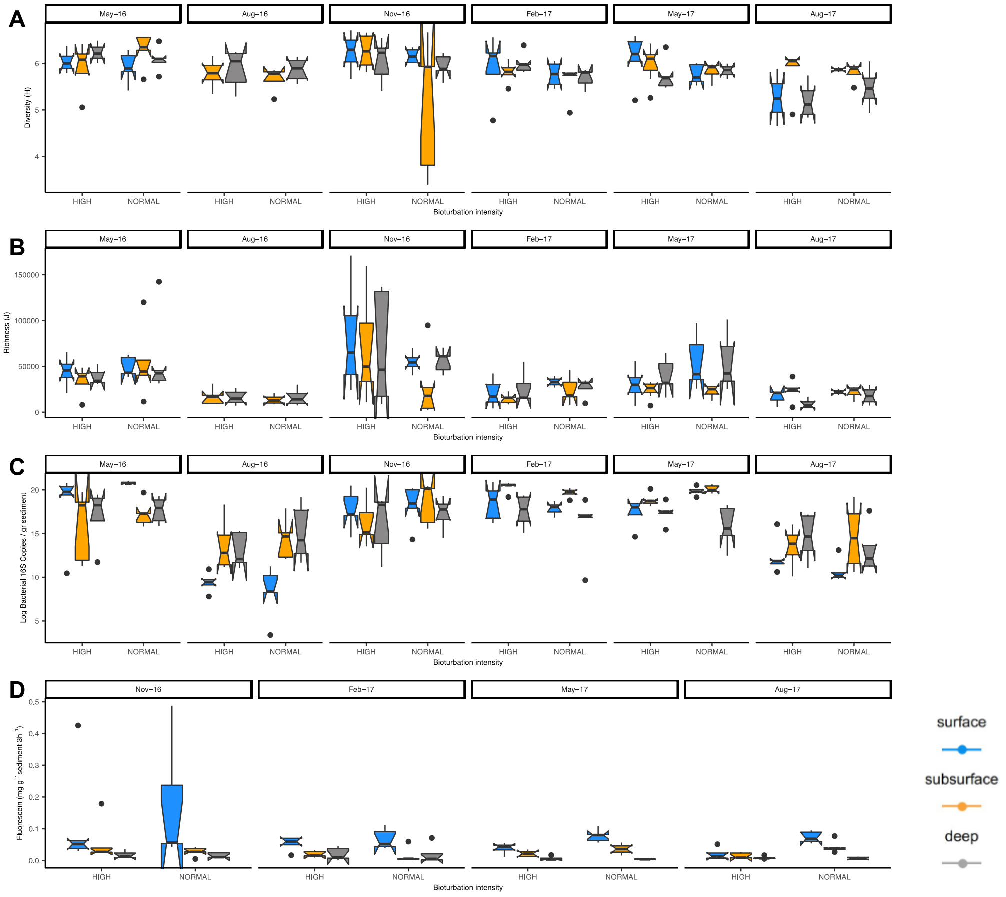

Supplementary Figure S5. Analysis of number of OTUs assigned to (A) photoautotrophy, (B) cellulolysis, (C) nitrate reduction, (D) nitrogen fixation, (E) sulfate respiration, (F) nitrification, (G) photoheterotrophy, and (H) sulphite respiration in relation to 'bioturbation intensity', 'depth' and 'month'.

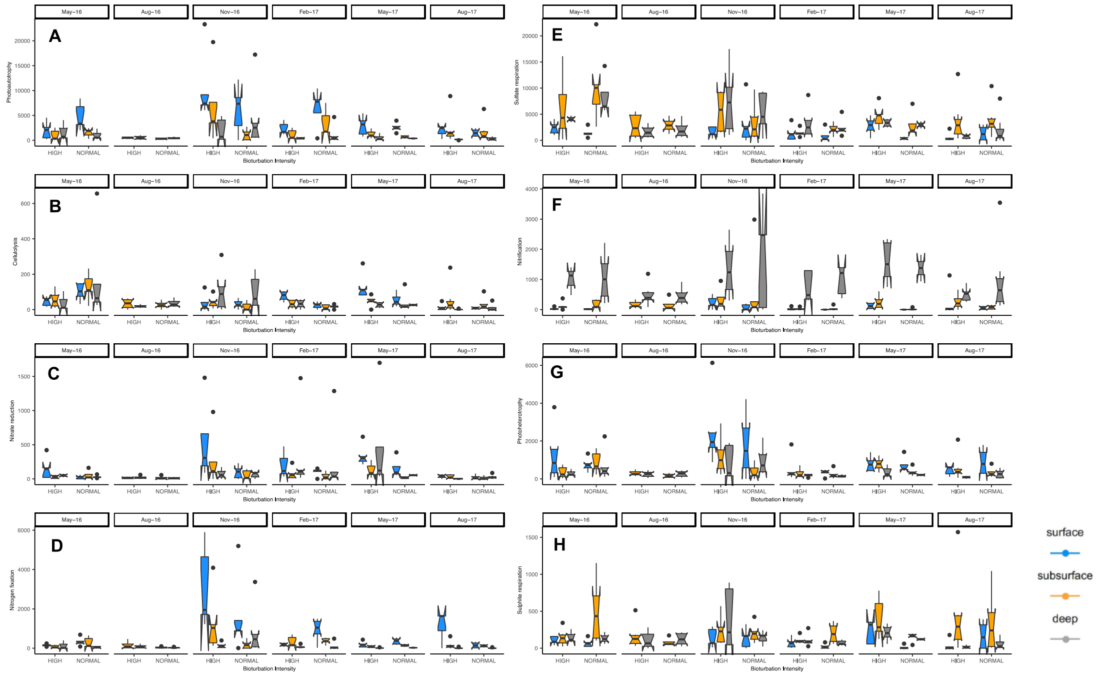

Supplement: Supplemental file 1 — Tables S1 and S2; Fig. S1 to S5. Download spectrum.01117-22-s0001.pdf, PDF file, 0.8 MB [file spectrum.01117-22-s0001.pdf]
